# Crude Extracts, Flavokawain B and Alpinetin Compounds from the Rhizome of *Alpinia mutica* Induce Cell Death via UCK2 Enzyme Inhibition and in Turn Reduce 18S rRNA Biosynthesis in HT-29 Cells

**DOI:** 10.1371/journal.pone.0170233

**Published:** 2017-01-19

**Authors:** Ibrahim Malami, Ahmad Bustamam Abdul, Rasedee Abdullah, Nur Kartinee Bt Kassim, Rozita Rosli, Swee Keong Yeap, Peter Waziri, Imaobong Christopher Etti, Muhammad Bashir Bello

**Affiliations:** 1 Laboratory of MAKNA-Cancer Research, Institute of Bioscience, Universiti Putra Malaysia Serdang, Selangor, Malaysia; 2 Department of Pharmacognosy and Ethnopharmacy, Faculty of Pharmaceutical Sciences, Usmanu Danfodiyo University, Sokoto, Nigeria; 3 Department of Biomedical Sciences, Faculty of Medicine and Health Sciences, Universiti Putra Malaysia, Selangor, Malaysia; 4 Department of Veterinary Pathology and Microbiology, Faculty of Veterinary Medicine, Universiti Putra Malaysia Serdang, Selangor, Malaysia; 5 Department of Chemistry, Faculty of Science, Universiti Putra Malaysia Serdang, Selangor, Malaysia; 6 Laboratory of Vaccine and Immunotherapeutics, Institute of Bioscience, Universiti Putra Malaysia Serdang, Selangor, Malaysia; 7 Department of Pharmacology and Toxicology, Faculty of Medicine and Health Sciences, Universiti Putra Malaysia Serdang, Selangor, Malaysia; Virginia Commonwealth University, UNITED STATES

## Abstract

Uridine-cytidine kinase 2 is an enzyme that is overexpressed in abnormal cell growth and its implication is considered a hallmark of cancer. Due to the selective expression of UCK2 in cancer cells, a selective inhibition of this key enzyme necessitates the discovery of its potential inhibitors for cancer chemotherapy. The present study was carried out to demonstrate the potentials of natural phytochemicals from the rhizome of *Alpinia mutica* to inhibit UCK2 useful for colorectal cancer. Here, we employed the used of *in vitro* to investigate the effectiveness of natural UCK2 inhibitors to cause HT-29 cell death. Extracts, flavokawain B, and alpinetin compound from the rhizome of *Alpinia mutica* was used in the study. The study demonstrated that the expression of UCK2 mRNA were substantially reduced in treated HT-29 cells. In addition, downregulation in expression of 18S ribosomal RNA was also observed in all treated HT-29 cells. This was confirmed by fluorescence imaging to measure the level of expression of 18S ribosomal RNA in live cell images. The study suggests the possibility of MDM2 protein was downregulated and its suppression subsequently activates the expression of p53 during inhibition of UCK2 enzyme. The expression of p53 is directly linked to a blockage of cell cycle progression at G0/G1 phase and upregulates Bax, cytochrome *c*, and caspase 3 while Bcl2 was deregulated. In this respect, apoptosis induction and DNA fragmentation were observed in treated HT-29 cells. Initial results from *in vitro* studies have shown the ability of the bioactive compounds of flavokawain B and alpinetin to target UCK2 enzyme specifically, inducing cell cycle arrest and subsequently leading to cancer cell death, possibly through interfering the MDM2-p53 signalling pathway. These phenomena have proven that the bioactive compounds could be useful for future therapeutic use in colon cancer.

## Introduction

Uridine-cytidine kinase 2 (UCK2) is an enzyme that catalyses the conversion of uridine and cytidine to their monophosphate form of uridine and cytidine in an alternative salvage pathway of pyrimidine biosynthesis [1]. A formation of 5^'^-triphosphate form of uridine and cytidine nucleosides are an essential requirement in gene replication. Overexpression of this enzyme have been implicated in several cancers and it is therefore considered a hallmark of cancer. The selective expression and non-immunogenicity of human UCK2 may, however represent a potential target for anticancer drug development [2].

Tumour suppressor protein, p53, prevents cancer development by eliminating cells with mutagenic alterations or potential for neoplastic transformation or blocking their cell cycle permanently or by transient DNA repair [3–5]. p53 is regulated by human double minute 2 (MDM2), an E3 ubiquitin ligase that targets and binds to p53 promoting ubiquitination and degradation of the protein [6,7]. Overexpression of MDM2 leads to inactivation of p53 tumour protein, thereby diminishing its tumour suppressor function [8]. Nonetheless, MDM2 is in turn regulated by ribosomal proteins (RPs) that binds and suppress the MDM2 E3 ubiquitin ligase activity resulting in the stabilization and activation of p53 [9]. These ribosomal proteins are found in stoichiometric amounts in the ribosome, thus, they are abundantly expressed in metabolically active cells undergoing protein synthesis [9,10]. The RPs are produced via a complex process comprising transcription, modification, processing of ribosomal RNA (rRNA) and subsequent production of these RPs [6]. Transcription of ribosomal DNA (rDNA) genes by RNA polymerase I produce the 47s rRNA precursor, and the modification and processing of this transcript, thus generate the mature 18S, 5.8S, and 28S rRNA. The rRNAs are assembled with the RPs to form 60S and 40S subunit of the mature ribosome [11,12]. The subunit of 60S contains 28S, 5.8S and 5S rRNAs, whilst the 40S subunit contains mainly 18S rRNA [13]. In response to the instability of ribosomal biogenesis such as the depletion of nucleotides, many RPs are released from the nucleolus and block MDM2 that targets p53 for degradation, this leads to p53 induction and cell cycle arrest [14].

In spite of the historical research in the area of cancer and its therapeutic targets, research in cancer has neglected the importance of UCK2 enzyme and its major role in the metabolic pathway of ribonucleotides biosynthesis required in gene synthesis as well as being a potential target for chemotherapy. Safety aspects in the use of newly marketed chemotherapeutic agents currently undergoing clinical trials against different types of cancer have been a worrisome due to severe adverse effects often accompanied with their use. Due to the serious side effects as evidenced by the use of synthetic ribonucleoside analogues, effective anticancer drugs of the natural origin, having less potential side effect in patients would then be highly desirable. To date, there has not been a single study conducted on any natural bioactive compound targeting the UCK2 enzyme. At this time of writing, no inhibitor from natural origin of this enzyme have been reported. This is the first report study of natural compounds that have the ability to inhibit the UCK2 enzyme.

Our laboratory has recently shown that phytocompounds flavokawain B (FKB) and alpinetin (APN) obtained from the rhizome of *Alpinia mutica* inhibits UCK2 enzyme *In Silico* [15]. We therefore tend to investigate further the mechanism by which FKB and APN inhibit growth of colorectal cancer through inhibition of UCK2 enzyme in human HT-29 cells.

## Materials and Methods

### Cell culture and propagation

The HT-29, HepaRG and Vero cell lines used in this study was obtained from American Type Culture Collections (ATCC) and was propagated in the laboratory of MAKNA Cancer Research of the Institute of Bioscience, Universiti Putra Malaysia. The cell line was sub-cultured in Dulbecco’s modified eagle’s medium (DMEM) media (Sigma-Aldrich, St. Louis, MO, USA) supplemented with 10% fetal bovine serum (FBS) (PAA, Freibug, Germany) and 1% 100 IU penicillin and 100 μg.mL^-1^ streptomycin (Sigma, USA). The starting culture was at 1 × 10^4^ cells.mL^-1^ and maintained at a temperature of 37°C in a humidified incubator containing 5% CO_2_. Cultures were continuously maintained by routine harvesting of cells at 70%–80% confluence using 0.05% trypsin-EDTA (Sigma-Aldrich, St. Louis, MO, USA).

### Extracts and phytocompounds from the rhizome of *A*. *mutica*

Crude hexane and chloroform extract, FKB and APN used in this investigation were obtained from the rhizome of *Alpinia mutica* [15]. The rhizomes (Voucher No. SK 3095/16) were obtained from the garden of the Institute of Bioscience, Universiti Putra Malaysia. A stock solution of both crude hexane and chloroform were prepared at a concentration of 10 mg in 50 μL dimethyl sulfoxide (DMSO) (Sigma-Aldrich, St. Louis, MO, USA), while FKB and APN were prepared at a concentration of 400 μM in 50 μL DMSO and the final concentration of DMSO will be 0.1% (*v*/*v*).

### Cell viability study

The effect of crude extracts and phytocompounds on HT-29 cells was examined using MTT assay. Briefly, cells were seeded in 96-well microplate at a density of 0.5 × 10^4^ cells/mL. Cells were treated after 24 h incubation at different concentrations in serial dilution with either crude extracts (200, 100, 50, 25, 12.5, and 6.25 μg/mL) or the phytocompounds (400 μM, 200 μM, 100 μM, 50 μM, 25 μM and 12.5 μM) for 72 h, respectively. 5-fluorouracil (400 μM, 200 μM, 100 μM, 50 μM, 25 μM and 12.5 μM) was used as a positive control while 0.1% (*v*/*v*) DMSO was used as negative control. After 72 h incubation, 20 μL of MTT stock solution (5 mg/mL) was added to each well and 100 μL of DMSO was added to each well after 4 h incubation in the dark. The amount of purple formazan formed was measured colorimetrically at 570 nm. Cell viability was expressed as the percentage of amount of viable cells to that of the amount of the total cell population and the potency of testing drugs to inhibit cell growth by 50% was expressed as IC_50_. The cell viability assay was carried out in three independent experiments.

### RT-PCR of mRNA expression

Total RNA from treated HT-29 cells was extracted using Aurum total RNA mini kit (Bio-rad, Hercules, CA, US) according to the manufacturer’s instructions. The total RNA was quantified using a nanodrop BioSpectrometer (Eppendorf, Hamburg, Germany). First strand cDNA was synthesised from total RNA using RevertAid cDNA synthesis kit (Thermo Scientific, Waltham, MA, US) according to the manufacturer’s instructions. Briefly, approximately 1 μg total RNA, random hexamer, 5× reaction buffer, RNase inhibitor, 10 mM dNTP, and RevertAid were mixed together. The total of 20 μL reaction volume was made with nuclease-free water and gently centrifuged. The mixture was incubated for 5 min at 25°C followed by 60 min at 42°C using a DNA thermal cycler (Eppindorf, Hamburg, Germany). The reaction was terminated for 5 min at 70°C to inactivate the reverse transcriptase and placed on ice at 4°C. A total of 20 μL reaction volume containing cDNA template was amplified using Exprime Taq premix (Genet Bio, Daejeon, Korea) according to the manufacturer’s instructions. The primer sequences used for PCR amplification are forward: 5^’^-GCGAACCATGGCCGGGGACAGCGAG-3^’^ and reverse: 5^’^-ACAGTATGTACAGATGAGCAGTGCC-3^’^ for UCK2, while GAPDH forward: 5'-TCCACCACCCTGTTGCTGTA-3^’^, and reverse 5'-ACCACAGTCCATGCCATCAC-3' was used as loading control.

### Fluorescence imaging of 18S RNA expression

SmartFlare RNA detection probe (SFST18S Hu-Cy5, Merck Millipore, Darmstadt, Germany) was used to measure the level of 18S RNA expression in HT-29 cells. Briefly, 196 μL each containing 2 × 10^3^ HT 29 cells were seeded in black 96-well microplate with clear bottom. After 24 h of incubation, HT-29 cells were treated with either crude extracts or phytocompounds at IC_50_ concentration, while DMSO at 0.1% (*v*/*v*) was used as a control. After 48 h post treatment, 4 μL (SmartFlare reagent 1:20 in sterile PBS) of the target SmartFlare probe was added to the treated HT-29 cells, while 4 μL of each scramble and uptake SmartFlare probe control was added to untreated HT-29 cells. Cells were incubated overnight (at least 16 h) at 37°C in a humidified incubator containing 5% CO_2_.

### Cell staining

Just before imaging, cell culture medium was gently removed and cells were washed with PBS for 3 consecutive times to ensure complete media removal. 200 μL of acridine orange (AO) in PBS from 1% stock concentration of AO (PromoKine, Heidelberg, Germany) was added into each well containing HT-29 cells and incubate at 37°C in a humidified incubator containing 5% CO_2_ for 15 min. The AO solution was removed, washed twice with PBS and immediately observed under fluorescence microscope. The fluorescent intensity was measured from the bottom using Zen lite (Carl Zeiss, Oberkochen, Germany). Cy5 fluorescence was measured with an excitation of 650 nm and an emission 670 nm, while AO was measured with an excitation of 493 nm and an emission 526 nm.

### Cell cycle analysis

The cell cycle analysis was performed using BD Cyclestest Plus DNA Kit (BD Biosciences, San Jose, CA, US) according to manufacturer’s instructions. Briefly, HT 29 cells was seeded in 6-well plate at a density of 5 × 10^5^ cells in each well and incubate at overnight 37°C in a humidified incubator containing 5% CO_2_ to allow adherence to the plate. The next day, cells were treated at different concentrations with 12.5, 25, and 50 μM of FKB and allowed to incubate for 72 h. On d 3, cells were harvested, washed with PBS and resuspended in buffer solution. The cells were centrifuged at room temperature for 5 min at 300 ×*g* and supernatant was carefully removed. 250 μL of solution A was added to each tube, mixed by gentle tapping and allowed to incubate for 10 min at room temperature. 200 μL of solution B was added to same tubes, mixed by gentle tapping and allowed to incubate for another 10 min at room temperature. 200 μL of ice cold solution C was added to the same tubes, mixed by gentle tapping and allowed to incubate in the dark on ice for another 10 min. HT-29 cells were directly analysed using BD flow cytometer (BD Biosciences, San Jose, CA, US).

### DNA fragmentation analysis

Briefly, DNA from treated HT-29 cells was extracted using Apoptotic DNA Ladder Kit (Roche, Basel, Switzerland) according to the manufacturer’s protocol. Approximately 10 μL of the extracted DNA was run on 1% gel electrophoresis and visualized under Gel Doc (Bio-rad, Hercules, CA, US).

### Extraction of total protein

Total protein was extracted from lysed HT-29 cells in an appropriate volume of ProteoJET mammalian cell lysis reagent (Fermentas, Burlington, ON, Canada). Cells were centrifuged at 22,000 ×*g* for 15 min and the supernatant was carefully collected, aliquots in PCR tubes and stored at −80°C until use. The total protein concentration in the cell lysate was estimated using Bradford reagent (Bio-Rad Laboratories, Hercules, CA, USA).

### Western blot analysis

A mixture of protein sample (25 μg) was separated by electrophoresis (Bio-rad, Hercules, CA, US) on an SDS-polyacrylamide gel followed by transfer of the separated proteins into a polyvinylidene difluoride membrane (Bio-rad, Hercules, CA, US) using semi-dry transblot Turbo (Bio-rad, Hercules, CA, US). The membrane was incubated for 1 h with 5% non-fat dry milk (Bio-rad, Hercules, CA, US) in TBS-T (20mM Tris-HCl (pH 7.5), 500mM NaCl, 0.05% Tween 20) to block nonspecific binding. Following incubation, membrane was washed with TBS-T and incubated for 2 h at room temperature with a labelled primary antibody. The target proteins were finally detected after labelling with horseradish peroxidase-conjugated IgG using ECL chemilumminescence in ChemiDoc (Bio-rad, Hercules, CA, US). The antibodies used in this investigation are: rabbit anti-UCK2 (Abnova, Taipei, Taiwan), mouse anti-β-Actin, rabbit anti-Bcl2, rabbit anti-Bax, rabbit anti-caspase-3 (Santa Cruz, Dallas, TX, US), rabbit anti-MDM2 (Acris, Herford, Germany), mouse anti-p53, mouse anti-Cyt *c*, horseradish peroxidase-conjugated Goat anti-mouse IgG, horseradish peroxidase-conjugated Donkey anti-mouse IgG (Biolegend, San Diego, CA, US).

### Statistical analysis

Results were expressed as mean ± SD for at least three replicate analyses for each sample. Statistical analysis was performed using GraphPad Prism 5.0. Analyses of variance were performed, followed by Dunnett’s and Bonferroni’s test for multiple comparison, whilst *p* values greater than 0.05 were considered significant.

## Results

### Crude extracts and bioactive compounds of FKB and APN inhibits proliferation of HT-29 cells

**Fig 1:**
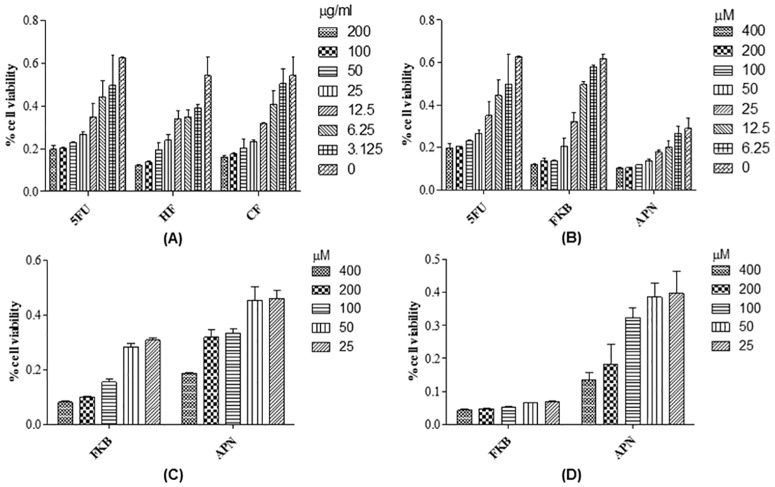
**Percentage cell viability of HT-29 cells treated with** (A) Different crude extract from the rhizome of *Alpinia mutica* at different concentrations (μg/mL) for 72 hrs (B) FKB and APN at different concentrations (μM) for 72 hrs (C) HepaRG cells treated with FKB and APN at different concentrations ((μM) for 72 hrs (D) Vero cells treated with FKB and APN at different concentrations (μM) for 72 hrs. MTT assay was used to determine the IC_50_ of the tested crude extract and phytocompounds.

The MTT colorimetric assay was used to measure the percentage cell viability of HT-29 cells treated either with the crude extracts or phytocompounds after 72 h incubation. The results showed that both hexane and chloroform extracts inhibit 50% cell proliferation at IC_50_ values of 21.05 μg/mL and 19.09 μg/mL, respectively (Fig 1A). On the other hand, HT-29 cells treated with FKB and APN showed 50% inhibition of cell proliferation at IC_50_ values of 27.68 μM (7.86 μg/mL) and 44.2 μM (11.17 μg/mL) respectively (Fig 1B). During this investigation, 5-Fluorouracil (5FU) was used as positive control in the study. HepaRG and Vero cells were later used in this study to determine if the bioactive compounds, FKB and APN have any effect towards normal cells. The results showed that treatment with FKB inhibit cell growth at IC_50_ values of 102.3 μM (29.03 μg/mL) and 189.08 (53.7 μg/mL) for the mammalian liver and kidney cells, respectively. Furthermore, cells treated with APN showed cell growth inhibition at IC_50_ values of 337.04 μM (140.80 μg/mL) and >400 μM (>113.6 μg/mL) for the liver and kidney cells, respectively (Fig 1C and 1D).

### Crude extracts containing bioactive phytocompounds responsible in downregulating the expression of UCK 2 enzyme

**Fig 2:**
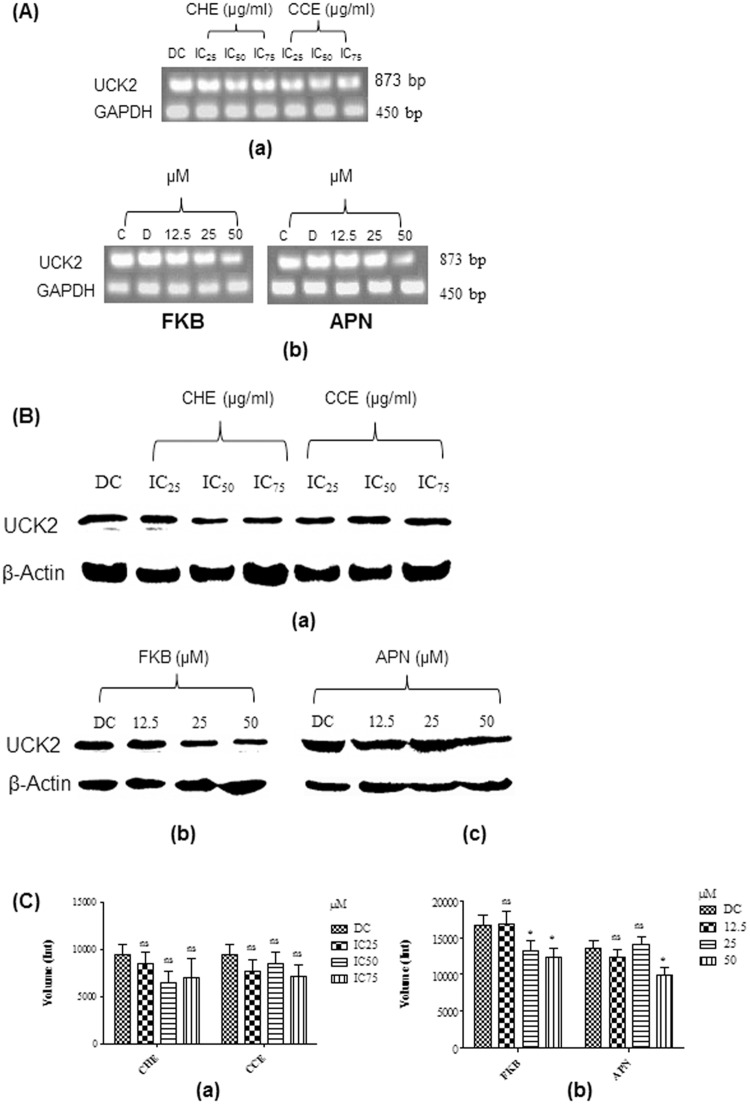
(A) Expression of UCK2 mRNA in HT-29 cells analysed in 1% agarose gel. (a)Levels of UCK2 mRNA expression in cells treated with increasing concentration of crude hexane (IC_25_: 10.52, IC_50_: 21.05, and IC_75_:42.1 μg/mL) and chloroform (IC_25_: 9.5, IC_50_: 19.09, and IC_75_:38.18 μg/mL) extracts; (b) Levels of UCK2 mRNA expressed in cells treated with FKB at 12.5 (3.55 μg/mL), 25 (7.1 μg/mL), and 50 μM (14.2 μg/mL); (c) Levels of UCK2 mRNA expressed in cells treated with APN at a concentration of 12.5 (3.37 μg/mL), 25 (6.75 μg/mL), and 50 μM (13.5 μg/mL). The housekeeping gene, GAPDH was used as loading control. C, Untreated control; D, DMSO used as negative control at a final concentration of 0.1%. (B) Western blot analysis of UCK2 protein expressed in HT-29 cells. (a) Levels of UCK2 protein expression in cells treated with increasing concentration of crude hexane (IC_25_: 10.52, IC_50_: 21.05, and IC_75_:42.1 μg/mL) and chloroform (IC_25_: 9.5, IC_50_: 19.09, and IC_75_:38.18 μg/mL) extracts. (b) Levels of UCK2 protein expressed in cells treated with FKB at a concentration of 12.5 (3.55 μg/mL), 25 (7.1 μg/mL), and 50 μM (14.2 μg/mL). (c) Levels of UCK2 protein expressed in cells treated with APN at a concentration of 12.5 (3.37 μg/mL), 25 (6.75 μg/mL), and 50 μM (13.5 μg/mL). (C) Levels of UCK2 protein expression quantified from western blotting analysis using Bio-rad Image Lab software in HT-29 cells treated with (a) Crude hexane and chloroform extract, and (b) Bioactive compounds of FKB and APN;. DC: DMSO treated control at a final concentration of 0.1%. Data are expressed as Mean±SD; ns: non-significant; *p<0.05; ns: non-significant compared to the DMSO control.

The level of UCK2 mRNA expressed in treated HT-29 cells was determined to examine if the crude extracts and the bioactive compounds of FKB and APN downregulate the activity of UCK2 enzyme. RT-PCR analysis showed significant decrease in the level of UCK2 mRNA in a dose dependent manner comparatively to DMSO as a control. The chloroform extract however, showed more preference to UCK2 activity in a dose dependent manner, but the intensity of the band obtained increased slightly at IC_75_. On the contrary, substantial decrease in the UCK2 mRNA expression in HT-29 cells treated with either FKB or APN compounds was observed in a dose dependent manner as compared to DMSO as control, strongly suggesting that UCK2 activity may possibly be inhibited (Fig 2A). To further substantiate our findings, Western blot analysis was used to investigate the expression of UCK2 proteins in HT-29 cells after 72 h of treatment with either the crude extracts or FKB and APN compounds. The study showed that the crude hexane extract at IC_50_ and IC_75_ concentrations is able to downregulate UCK2 protein levels in HT-29 cells, whilst similar expression of downregulation of UCK2 was also observed in HT-29 cells treated with chloroform extract. Similarly, levels of UCK2 protein expressed was downregulated after 72 h treatment with either FKB or APN. The analysis showed that FKB and APN were able to inhibit the expression of UCK2 protein most likely at 50 μM (Fig 2B).

### Crude extracts containing the bioactive phytocompounds downregulates expression of 18S RNA in live cell images

**Fig 3:**
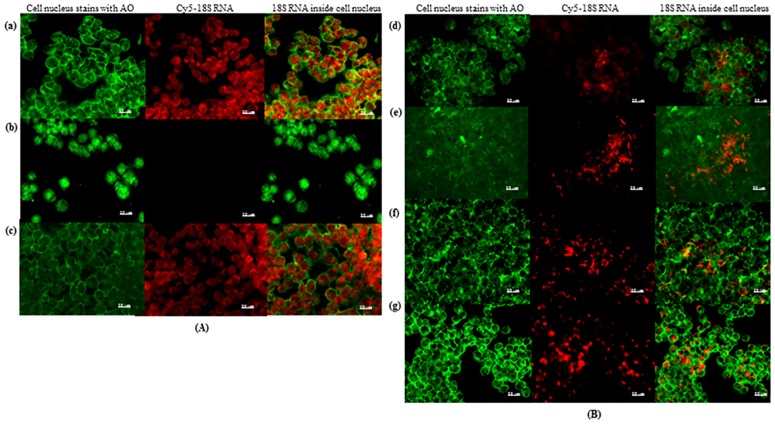
(A) Live cell imaging of the controls of HT-29 cells with SmartFlare Cy5-Uptake, Cy5-Scramble or Cy5-18S probes, imaged using fluorescence microscope in exposure settings at 20× magnification. (a) Uptake probe used as positive control that quenched on inside the cell. (b) Scramble probe used as negative control where the probe is not able to recognize any RNA sequence inside the cell nucleus. (c) Cells with 18S probe after 48 h of 0.1% (*v*/*v*) DMSO treatment as negative control. (B) Live cell imaging of HT-29 cells with SmartFlare Cy5-18S probes, and imaged using fluorescence microscope in exposure settings at 20× magnification. Cells with 18S probe after 48h treatment with crude extract of (d) hexane extract (21.05 μg/mL), (e) chloroform extract (19.09 μg/mL), (f) FKB (8.47 μg/mL), and (g) APN (13.12 μg/mL).

Live cell imaging detection of 18S RNA in treated and non-treated HT-29 cells using SmartFlare Cy5-18S nanoprobe was used to determine whether downregulation of UCK2 enzyme could possibly interfere with synthesis of 18S RNA. HT-29 cells were treated initially with Cy5-Uptake probes (probe that quenched on inside the cell) and Cy5-Scramble probes (probe does not recognise any RNA sequence inside the cell) in order to ensure the effectiveness of the SmartFlare probes used in the analysis. The Cy5-Uptake control probe provides more intense fluorescence compared to the Cy5-Scramble control probe after an overnight incubation (Fig 3A). Consequently, treated HT-29 cells containing Cy5-18S probe examined under fluorescence microscopy showed micrographs of gradually decreased in the synthesis of 18S RNA at IC_50_ concentration (Fig 3B). Comparatively, the fluorescent intensity of Cy5-18S probe in HT-29 cells treated with either the crude extracts or FKB and APN compounds was intensively reduced when comparatively compared to the DMSO as a control. This strongly indicates that the Cy5-18S probe has the capability to recognise 18S RNA inside the HT-29 cells after overnight incubation with the probes. This reduction in 18S rRNA synthesis after treatment of HT-29 cells with either crude extracts or FKB and APN compounds denotes the reduction to the biosynthesis of nucleotides of the cell’s nucleus.

### Defects in 18S rRNA synthesis induced by crude extracts, FKB and APN compounds may have distinctive correlation to the suppression of MDM2 bound to p53

**Fig 4:**
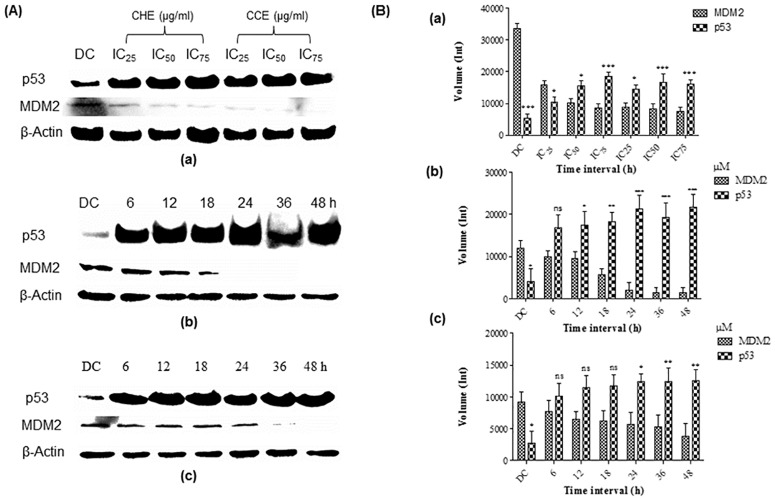
Levels of MDM2 and p53 proteins expressed in HT-29 cells. (A) Level of proteins in cells treated with (a) crude hexane (IC_25_: 10.52, IC_50_: 21.05, and IC_75_:42.1 μg/mL) and chloroform (IC_25_: 9.5, IC_50_: 19.09, and IC_75_:38.18 μg/mL) extracts. (b) 25 μM (7.1 μg/ mL) of FKB at different time interval. (c) 25 μM (6.75 μg/mL) of APN at different time interval. (B) Level of MDM2 and p53 protein expression quantified from western blotting analysis using Bio-rad Image Lab software in HT 29 cells treated with (a) Hexane and chloroform extracts (b) FKB, and (c) APN. Data are expressed as Mean±SD; ns: non-significant; *p<0.05; **p<0.01; ***p<0.01; ns: non-significant compared to the DMSO control. DC: DMSO used as negative control at a final concentration of 0.1%.

The above parameter was investigated using Western blotting of proteins in HT-29 cells treated at various concentrations (IC_25_, IC_50_, and IC_75_) of bioactive crude extracts to examine the levels of MDM2 and p53 protein expressions after 72 h treatment. The analysis demonstrated that the expression MDM2 protein was substantially downregulated in a dose dependent manner compared with control. Concurrently, expression of p53 protein was clearly observed to increase in a dose dependent manner compared to control (Fig 4A and 4B). In respect of these results, expression of MDM2 protein treated with FKB at 25 μM (7.1 μg/ mL) or APN at 25 μM (6.75 μg/ mL) was found to be substantially downregulated compared with DMSO treated control and was further undetected after 24 h incubation when using FKB and 36 h when using of APN. Meanwhile, expression of p53 protein was clearly expressed with increased to the protein levels in a time dependent manner compared to DMSO as a control (Fig 4A and 4B). The above results seem to suggest the expression p53 in treated HT-29 cells, whether with crude extracts or FKB and APN compounds, increased in a time dependent manner with subsequent decreased in MDM2 expression. The increased in p53 expression of treated HT-29 cells is well correlated with initial findings that implicates apoptosis and subsequently to cell death of HT-29 cells.

### Stabilization of p53 induced p53-dependent mitochondrial pathway in HT-29 cells

**Fig 5:**
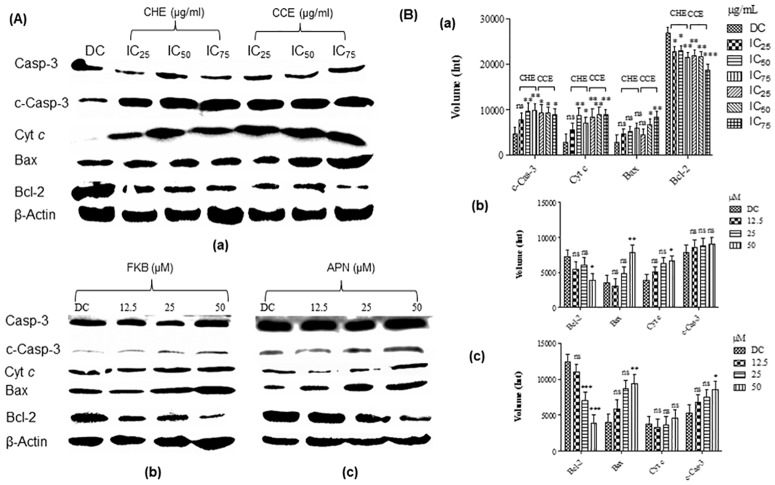
Levels of protein expression involved in mitochondrial apoptotic signalling pathway in HT-29 cells after 72 h incubation. (A) Levels of protein expressed in cells treated with (a) crude hexane (IC_25_: 10.52, IC_50_: 21.05, and IC_75_:42.1 μg/mL) and chloroform (IC_25_: 9.5, IC_50_: 19.09, and IC_75_:38.18 μg/mL) extracts. (b) FKB at a concentration of 12.5 (3.55 μg/mL), 25 (7.1 μg/mL), and 50 μM (14.2 μg/mL) and (c) APN at a concentration of 12.5 (3.37 μg/mL), 25 (6.75 μg/mL), and 50 μM (13.5 μg/mL). (B) Levels of protein expression quantified from western blotting analysis using Bio-rad Image Lab software in HT-29 cells treated with (a) Hexane and chloroform extracts (b) FKB, and (c) APN. Data are expressed as Mean±SD; ns: non-significant; *p<0.05; **p<0.01; ***p<0.001 ns: non-significant compared to the DMSO control. DC: DMSO treated control at a final concentration of 0.1%.

Western blot analysis was performed in this investigation to examine levels of related proteins which may be implicated in the apoptotic signalling pathway, leading to cell death. As shown in Fig 5A, HT-29 cells treated with the bioactive crude extract substantially downregulate expression of Bcl-2 in a dose dependent manner. On the contrary, expression of Bax at significant amount was observed in HT-29 cell treated with chloroform extract at a concentration of IC_50_ (19.09 μg/mL) and IC_75_ (38.18 μg/mL). In addition, Cyt *c* expression and caspase-3 cleavage was further observed to increase in a dose dependent manner after 72 h treatment with crude extracts. On the other hand, HT-29 cells treated with FKB in increasing concentration substantially downregulate the expression of Bcl-2 while Bax expression was upregulated in a dose dependent manner. In addition, Cyt *c* expression and caspase-3 cleavage was also observed to increase substantially in a dose dependent manner after 72 h treatment with FKB. Further to this, the study revealed that APN also induced the expression of Bax but downregulate the expression of Bcl-2 in a dose dependent manner. The levels of Cyt *c* expression and caspase-3 cleavage was also found to increase. This, confirms apoptosis induction was triggered by both bioactive compounds during treatment of HT-29 cells.

### Stabilization of p53 induced cell cycle arrest in HT-29 cells

**Fig 6:**
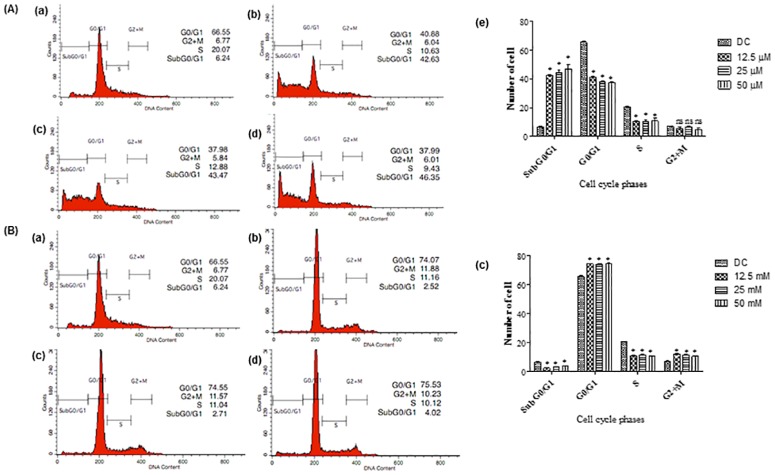
Cell cycle analysis examined using flow cytometry on HT-29 cells after 72 h treatment. (A) Cells treated with (a) DMSO at the final concentration of 0.1%. (b) FKB at a concentration of 12.5 (3.55 μg/mL), (c) 25 (7.1 μg/mL), (d) 50 μM (14.2 μg/mL), and (e) Percentage of cell cycle distribution in different phases. (B) Cells treated with (a) DMSO at the final concentration of 0.1%. (b) APN at 12.5 (3.37 μg/mL), (c) 25 (6.75 μg/mL), (d) 50 μM (13.5 μg/mL) concentrations, and (e) Percentage of cell cycle distribution in different phases. G0/G1, G2+M, and S are cell phases, respectively; subG0/G1 refers to cell death due to DNA fragmentation. Data are expressed as Mean±SD of three independent experiments, *p<0.001, ns: non-significant compared to the normal control.

The present findings clearly suggest that accumulation of p53 is primarily caused by the suppression of MDM2 through the disruption of ribosomal biogenesis in the cell’s nucleus. To further verify this, the consequence of p53 suprainduction induction on cell cycle progression of treated HT-29 cells was examined. Cell cycle analysis was performed to examine the HT-29 cell cycle progression treated with either FKB or APN compound. This study showed that HT-29 cells treated at different concentrations of FKB after 72 h induced the accumulation of dead cells at subG0/G1 phase in a dose dependent manner concurrently to DMSO treated control with significantly decreased in treated HT-29 cells at G0/G1 and S phase being observed (Fig 6A). On the other hand, HT-29 cells treated at different concentrations of APN blocked cell cycle progression at G0/G1 phase in a dose dependent manner, compared to DMSO treated control. Further to this, increased percentage of HT-29 cells were also found to accumulate at G2/M phase when treated at different concentrations of APN (Fig 6B).

### Bioactive compounds of FKB and APN induced apoptosis in turn triggering DNA fragmentation in treated HT-29 cells

**Fig 7:**
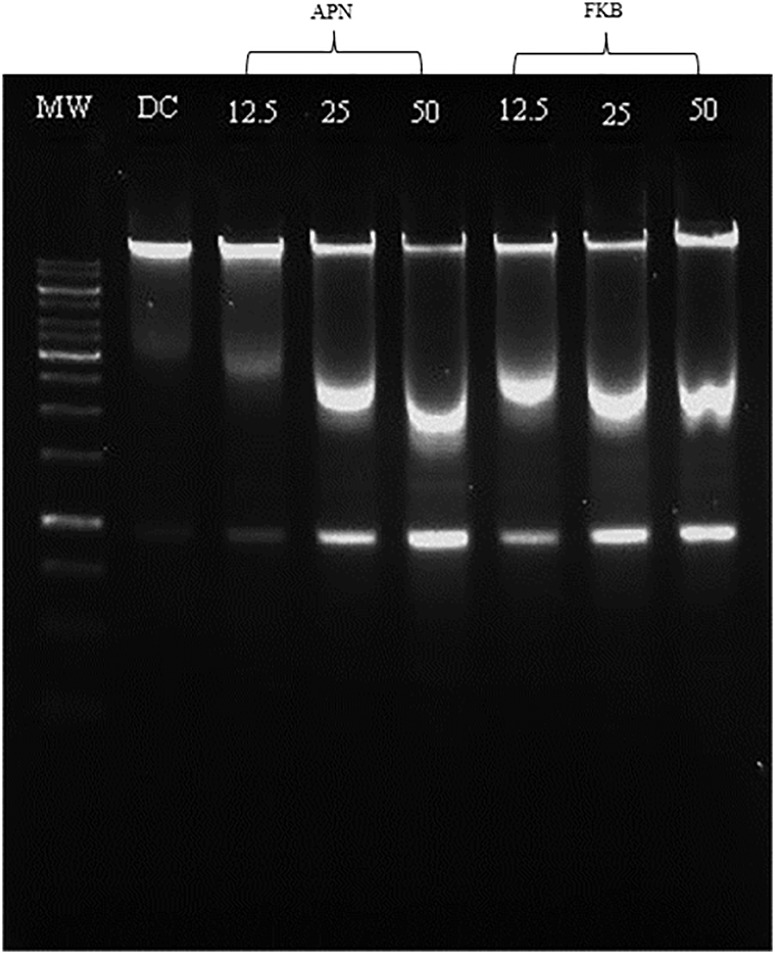
DNA fragmentation analysed in 1% agarose gel after 72 h incubation with different concentration of either FKB or APN. MW: DNA marker; DC: DMSO treated control; all concentrations are in μM.

DNA laddering was performed to further verify and confirm apoptosis induction HT-29 cells treated with either FKB or APN. Fig 7 showed DNA fragmentation after 72 h incubation at different concentrations with either FKB or APN. DNA fragmentation was obvious in cells treated with FKB in a dose dependent manner compared to DMSO treated cells concurrently. Furthermore, HT-29 cells treated with APN showed clear evidence of apoptosis induction after treatment with 25 and 50 μM concentrations compared to DMSO treated control. The appearance of ladder-like bands suggest the possibility of DNA fragmentations to occur when both bioactive compounds of FKB and APN were used to treat HT-29 cells. The appearance of the bands substantially becomes prominent as incubation increased to 72 h, suggesting it is time-dependant.

## Discussion

Interest in the search for potential natural anticancer for treatment has led to the discovery of UCK2 inhibitors from the rhizome of *Alpinia mutica*. Previous literatures have reported that the isolated bioactive compounds from the rhizome possess strong anticancer activities, but none to date have been considered for further development into chemotherapeutic agents. The current investigation provided insight to the isolated compounds, FKB and APN as potential UCK2 inhibitors for future treatment of colon cancer.

The study of cell viability in the present investigation verified the ability of the bioactive compounds to inhibit 50% of HT-29 cells at low concentrations. A previous study had reported that FKB inhibited proliferation of HCT 116 cells at an IC_50_ of 25 μM [16], which is consistent with the present finding. Moreover, FKB has been shown to inhibit 50% cell growth of SK-LMS-1 and 143B cells at a very low concentration of 2.2 μM (1.25 μg/mL) and 1.97 μM (3.5 μg/mL), respectively [17,18]. These IC_50_ values are, however 6 fold lower than that observed in colorectal cancer (HT-29 and HCT 116 cells), which in fact, is within acceptable concentration limits. In one related study, inhibition of two breast cancer cells by FKB at an IC_50_ of 12.3 and 33.8 μM in MDA-MB231 and MCF-7, respectively were reported [19]. APN on the other hand, have been previously reported to inhibit HCT 116 cells at an IC_50_ of 39.6 μg/mL [20]. This value is about 2 fold higher than that observed in the current findings.

Apart from the above, the present finding revealed that FKB is also cytotoxic towards normal liver and kidney cells tested on human HepaRG and Vero cells, respectively. The IC_50_ was determined to be 102.3 μM (29.03 μg/mL) and 189.08 (53.7 μg/mL) for the liver and kidney cells, respectively. These results, suggest that FKB is hepatotoxic and nephrotoxic towards normal liver and kidney cells. Earlier, Zhou and coworker, 2010, have shown that, the hepatocellular toxicity of FKB is the result of oxidative stress induced by the compound leading to the depletion of glutathione (GSH) both *in vitro* and *in vivo*. Thus, IκB kinase (IKK) activity is inhibited, leading to the blockage of NF-κB transcription, in turn, to the constitutive TNF-α-independent activation of mitogen-activated protein kinase (MAPK) signalling pathway. However, it was observed that replenishment with exogenous GSH normalizes both TNF-α-dependent NF-κB and MAPK signalling pathway, thus prevents normal liver and kidney cells from being damaged [21]. Indeed, this approach could be useful when using the FKB compound in treating cancer patients.

RT-PCR analysis was used to determine levels of UCK2 mRNA expressions in HT-29 cells. Quantification of UCK2 mRNA expressions in HT 1080, NUGC 3, NCI H630, AZ 521, DLD 1, MCF 7, and BxPC 3 cells have previously been reported elsewhere using this technique [22,23]. In this present study, levels of UCK2 mRNA expression were quantified in HT-29 cells using a similar protocol technique reported previously. The results demonstrated clearly that downregulation of UCK2 mRNA expressions were conclusive in all HT-29 treated cells with either the extracts or bioactive compounds of FKB and APN. Apparently, this is consistent with the western blotting analysis of UCK2 protein expressed in HT-29 cells treated with the bioactive compounds of FKB and APN initially. Statistically, there is significantly decreased to the levels UCK2 protein expressed in HT-29 cells after 72 h treatment with 25 and 50 μM of FKB, while, significant decreased was observed only at 50 μM of APN S1 Fig.

DMSO has been shown to greatly reduce synthesis of 18S rRNA in HL-60 leukemia cells [24]. Hence, DMSO at a final concentration of 0.1%, used as a vehicle, was examined if it had any effect on mRNA expression in HT-29 cell. The results from this investigation shows that neither changes in the levels of UCK2 nor 18S rRNA mRNA expressions were observed in 0.1% DMSO.

Nucleotides and nucleoside triphosphates such as guanine, guanosine 5'-triphosphate, and adenosine 5'-triphosphate have been shown to play a major role in rRNA synthesis inhibition [25,26]. The results of the RT-PCR and western blot analysis obtained in this investigation seem to support that downregulation of UCK2 mRNA expression consequently reduced synthesis of 18S rRNA in treated HT-29 cells. This may further suggest that the depletion of nucleotide biosynthesis through the inhibition of UCK2 may possibly lead towards downregulation of 18S rRNA mRNA expression in treated HT-29 cells with either the extracts or bioactive compounds of FKB and APN.

Defects in ribosomal biogenesis have previously been shown to activate p53 signaling pathway [27,28]. The MDM2-p53 signaling pathway has been an important regulator of cellular homeostatic [6]. In response to nucleolar stress via instability of ribosomal biogenesis, ribosomal proteins (RPs) such as RPS7 find their way and bind to MDM2 and block MDM2-mediated p53 ubiquitination and degradation, hence, p53 is activated which in turn induced cell cycle arrest and apoptosis induction [14]. Studies have shown that a selective inhibition in 18S rRNA synthesis activates the p53 signalling pathway [28]. In relation to this, the current investigation indicates clearly here that the inhibition of 18S rRNA at transcription level induced by either the bioactive crude extracts or bioactive compounds of FKB and APN, stabilizes p53 through interference with MDM2-p53 signalling. This is possible through RPS7 bound to MDM2 since the mature 40S subunit of ribosomes contains 18S rRNA [27]. Depletion in MDM2 expression has previously shown to contribute to the accumulation of p53 protein [29–31]. In order to determine if activation of p53 is MDM2-dependent inactivation, levels of MDM2 and p53 expressed in treated HT-29 cells were investigated at different time interval. The results showed that MDM2 protein expression was significantly downregulated with concomitant increased in p53 protein expression in a time-dependant manner following treatment with either the crude extract or the bioactive compounds of FKB and APN. The results of this investigation suggest clearly that the activation and stabilization of p53 is, in fact MDM2-dependent inactivation.

Many studies have previously shown that increase in the expression of p53 protein upregulate the expression of p53 downstream protein, MDM2, indicating intact p53 signalling [31–34]. In response to agent inducing stress, MDM2 play a crucial role in the stabilisation and activation of p53 signalling by increasing expression of protein synthesis via disrupting the MDM2-p53 interaction, this explains as to why MDM2 is overexpressed, thus indicates an intact p53 downstream signalling [33]. Similarly, p53 stabilisation have also been demonstrated in cancer cells expressing R273H mutant p53 such as HT-29 following treatment with various agents. The induction of cell death of cancer expressing R273H mutant p53 is either by restoring the wild-type p53 activity leading to its stabilisation [35–42] or by restoring sequence-specific DNA binding of mutant p53 [43,44], or by depleting mutant p53 with minimal effect on the wild-type p53 [45–51]. In the present study, the ability of the bioactive compounds of FKB and APN to specifically influence the disruption of MDM2-p53 complex, thereby causing direct activation of p53, which is essential in triggering cell cycle arrest and apoptosis in HT-29 cells was demonstrated. Although, we have shown that the ability of FKB and APN to cause depletion of MDM2 protein play an important role in the stabilisation and activation of p53 signalling in HT-29 cells expressing R273H mutant p53. However, an insight into the molecular mechanism underlying restoration of wild-type p53 activity in the present study is still not clear.

Western blot analysis of protein expression suggests that the bioactive compounds of FKB and APN induced p53 dependent apoptosis in treated HT-29 cells. Previous report has shown that caspase-9 and Apaf-1, which are basic proteins involved in mitochondrial pathway of apoptosis are found to be the essential downstream components of p53 in Myc-induced cell death [52]. The proapoptotic Bax protein, however, usually accumulates in the mitochondria in response to the death signals [53]. Moreover, the Bax gene promoter contains motifs with homology to p53-binding site and was earlier shown to response to p53 upon binding [54]. This proapoptotic Bax protein is critical in increasing permeability of mitochondrial membranes, thus implicated in the release of cytochrome c, which in turn, initiates a cascade of caspase activation leading to cell death [55]. Apparently, the present findings, clearly demonstrated that Bcl-2 expression was statistically downregulated, whilst Bax, and Cyt *c* expression were upregulated. Though, showing statically non-significant to the levels of c-Caspase 3 in HT-29 cells treated with FKB, the levels of Cyt *c* in HT-29 cells treated with APN, however, together with the western blotting profile demonstrated both proteins mentioned were upregulated at dose dependent manner. In this respect, it therefore could be suggested that the induction of apoptosis triggered by the bioactive compounds of FKB and APN is p53-dependent towards the HT 29 colon cancer cells.

Previous study conducted had shown that FKB induced cell cycle arrest at G2/M phase in HCT116 [16], SK-LMS-1 and ECC-1 [17], 143B and SaOS-2 [18], and MDA-MB231 and MCF-7 [19]. On the contrary, findings in this current investigation seem to suggest that increasing dead cells accumulated at subG0/G1 phase instead, rather than blocking cell progression at G2/M phase. Cell cycle arrest at G2/M phase in this current study is almost undetectable. Accumulation of dead cells at the subG0/G1 phase is often regarded as a marker of apoptosis, characterized by loss of DNA content as a result of fragmentation to their genetic DNA [56]. This is clearly observed as shown in the DNA fragmentation analysis of cells treated HT-29 cells. This DNA fragmentation phenomenon was obvious in HT-29 cells treated with either FKB or APN. HT-29 cells were further examined under fluorescence microscope for confirmation of apoptosis. Morphological changes clearly observed in HT-29 treated cells confirmed apoptosis comparatively to intact and healthy nuclear structure used as control S2 and S3 Figs.

The results obtained in this current investigation suggest the possibility of p53 activation prior to the triggering of apoptosis in treated HT-29 cells by FKB and APN compounds. This activation of p53 is in fact governed by the inhibition of the UCK2 enzyme and cell cycle arrest, in turn, creating events related towards activating a cascade of related proapoptotic proteins primarily involved in the signalling of mitochondrial pathway of apoptosis induction. All these have been attributed to the abilities of FKB and APN compounds in targeting specifically to the UCK2 enzyme. This study has revealed, for the first time, the capabilities of FKB and APN in the selective targeting of proteins implicated in arresting cancer cells from proliferating. The key to this selective targeting is the selective inhibition of the UCK2 enzyme, which has proven to be the main targeted protein in this cancer therapeutic.

The current investigation further revealed a descriptive declined in the 18S RNA biosynthesis, concomitantly implicating cell cycle arrest being encountered when UCK2 enzyme was inhibited. This correlation in the declined of 18S RNA biosynthesis to the cell cycle arrest suggest a strong possibility of nucleotide synthesis inhibition, which supports the suggestion of DNA breakdown, resulting towards DNA fragmentation as cellular signalling in triggering apoptosis inductions continues. The aftermath, is cell death of the cancer cells undergoing treatment, which reaffirms a strong possibility in using FKB and APN compounds as the future cancer therapeutics for colon cancer.

## Conclusion

In conclusion, we have shown for the first time that FKB and APN act as potential UCK2 inhibitors *in vitro*. The ability of the crude extracts to inhibit the UCK2 enzyme denotes possible presence of compounds in the extracts that are able to bind towards binding sites of the enzyme. These compounds may possibly possess stereochemistry and molecular structures near similar or parallel to FKB or APN. Likewise, the presence of these compounds that mimics parallel mechanism of action to FKB and APN in inhibiting the UCK2 enzyme, which subsequently induces apoptosis in a similar manner to suggest strongly that it may be possibly be this presence of FKB and APN compounds in the extracts that contributed to the anticancer properties of the crude extracts. The results obtained in using FKB and APN have clearly demonstrated the ability of these compounds in inhibiting this key enzyme responsible for the biosynthesis of 18S RNA. This inhibition using FKB and APN to the UCK2 enzyme may provide useful information as drug leads for future development of anticancer compounds. In addition, inhibition of the UCK2 enzyme activity by FKB and APN exhibited a strong correlation to the MDM2-p52 signalling pathway, in which current investigations have demonstrated the ability of FKB and APN in the destruction of the MDM2-p53 complex, in turn, being the cause of the activation of p53, which is essential in triggering cell cycle arrest and apoptosis induction in the cancer cells. Incidentally, the ability of FKB and APN compounds in targeting UCK2 specifically perhaps induces cell cycle arrest initially and later apoptosis induction via direct intervention to the MDM2-p53 pathway. The current results substantially have potential proven beyond doubt, FKB and APN have the potential to be compounds useful for future cancer therapeutic usage that warrants further development towards human clinical trials.

## Supporting Information

S1 Fig Expression of UCK2 mRNA in HT-29 cells analysed in 1% agarose gel. (A)Levels of UCK2 mRNA expression in cells treated with increasing concentration of crude hexane (IC_25_: 10.52, IC_50_: 21.05, and IC_75_:42.1 μg/mL) and chloroform (IC_25_: 9.5, IC_50_: 19.09, and IC_75_:38.18 μg/mL) extracts; (B) Levels of UCK2 mRNA expressed in cells treated with FKB at 12.5 (3.55 μg/mL), 25 (7.1 μg/mL), and 50 μM (14.2 μg/mL); (C) Levels of UCK2 mRNA expressed in cells treated with APN at a concentration of 12.5 (3.37 μg/mL), 25 (6.75 μg/mL), and 50 μM (13.5 μg/mL). The housekeeping gene, GAPDH was used as loading control. C, Untreated control; D, DMSO used as negative control at a final concentration of 0.1%.
(TIF)

S2 Fig Morphological examination of HT-29 cells treated with crude hexane extract at IC_25_: 10.52, IC_50_: 21.05, and IC_75_:42.1 μg/mL. Cells were stained with AO and imaged using fluorescence microscope in exposure settings at 20× magnification. DC: DMSO treated control at a final concentration of 0.1%.
(TIF)

S3 Fig Morphological examination of HT-29 cells treated with crude chloroform extract (IC_25_: 10.52, IC_50_: 21.05, and IC_75_:42.1 μg/mL), FKB at 12.5 (3.55 μg/mL), 25 (7.1 μg/mL), and 50 μM (14.2 μg/mL); APN at a concentration of 12.5 (3.37 μg/mL), 25 (6.75 μg/mL), and 50 μM (13.5 μg/mL). Cells were stained with AO and imaged using fluorescence microscope in exposure settings at 20× magnification. DC: DMSO treated control at a final concentration of 0.1%.
(TIF)
