# Correction: Crude Extracts, Flavokawain B and Alpinetin Compounds from the Rhizome of *Alpinia mutica* Induce Cell Death via UCK2 Enzyme Inhibition and in Turn Reduce 18S rRNA Biosynthesis in HT-29 Cells

**DOI:** 10.1371/journal.pone.0173651

**Published:** 2017-03-07

**Authors:** Ibrahim Malami, Ahmad Bustamam Abdul, Rasedee Abdullah, Nur Kartinee Bt Kassim, Rozita Rosli, Swee Keong Yeap, Peter Waziri, Imaobong Christopher Etti, Muhammad Bashir Bello

The affiliation for the ninth author is incorrect. Muhammad Bashir Bello is not affiliated with #7 but with #6, Laboratory of Vaccine and Immunotherapeutics, Institute of Bioscience, Universiti Putra Malaysia Serdang, Selangor, Malaysia

There are errors in Fig 6. The last graph in Fig 6(B) should be labeled (e). The y-axis in both Fig 6(A)(e) and Fig 6(B)(e) should read, “Number of cells (%).” Please see the corrected Fig 6 here.

**Fig 6:**
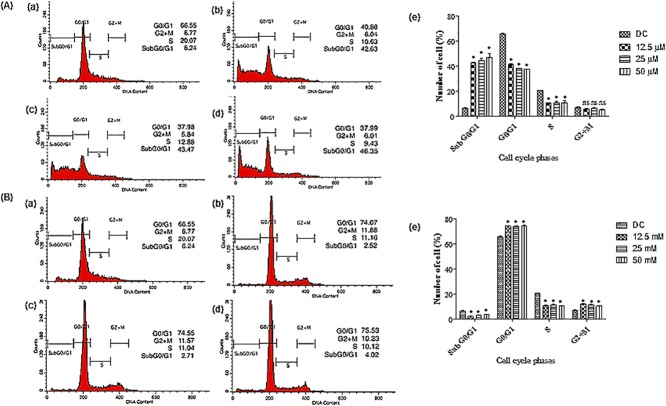
Cell cycle analysis examined using flow cytometry on HT-29 cells after 72 h treatment. (A) Cells treated with (a) DMSO at the final concentration of 0.1%. (b) FKB at a concentration of 12.5 (3.55 μg/mL), (c) 25 (7.1 μg/mL), (d) 50 μM (14.2 μg/mL), and (e) Percentage of cell cycle distribution in different phases. (B) Cells treated with (a) DMSO at the final concentration of 0.1%. (b) APN at 12.5 (3.37 μg/mL), (c) 25 (6.75 μg/mL), (d) 50 μM (13.5 μg/mL) concentrations, and (e) Percentage of cell cycle distribution in different phases. G0/G1, G2+M, and S are cell phases, respectively; subG0/G1 refers to cell death due to DNA fragmentation. Data are expressed as Mean±SD of three independent experiments, *p<0.001, ns: non-significant compared to the normal control.
