# Supplementary figures and images for: Crude Extracts, Flavokawain B and Alpinetin Compounds from the Rhizome of Alpinia mutica Induce Cell Death via UCK2 Enzyme Inhibition and in Turn Reduce 18S rRNA Biosynthesis in HT-29 Cells
Source: PLoS One. 2017 Jan 19;12(1):e0170233. doi: 10.1371/journal.pone.0170233 (PMC5245823; doi:10.1371/journal.pone.0170233)

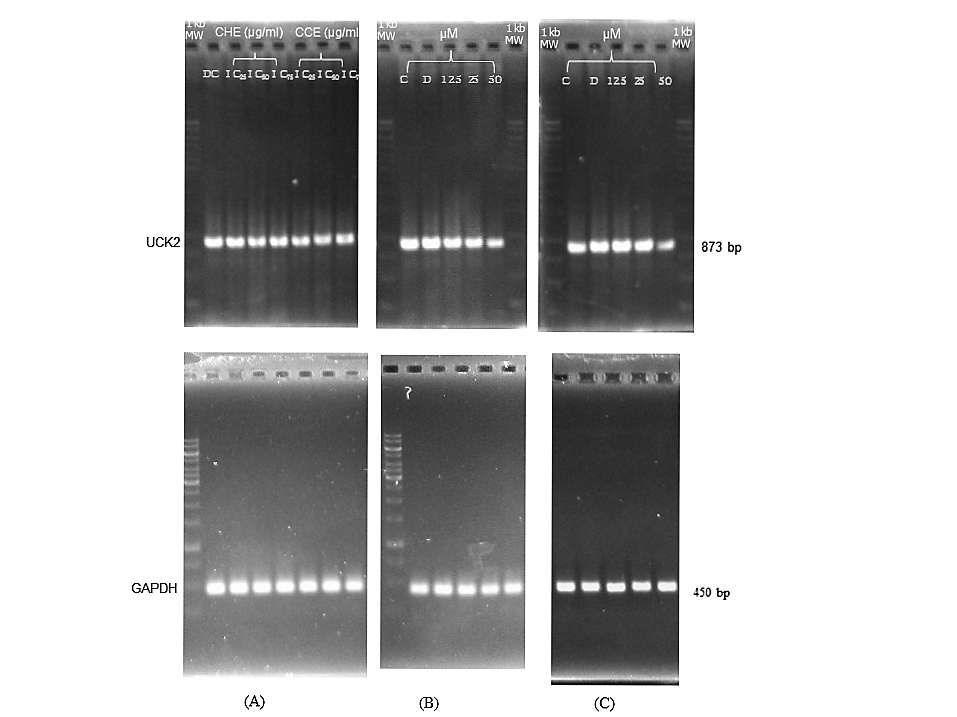

Supplement: S1 Fig — (A)Levels of UCK2 mRNA expression in cells treated with increasing concentration of crude hexane (IC25: 10.52, IC50: 21.05, and IC75:42.1 μg/mL) and chloroform (IC25: 9.5, IC50: 19.09, and IC75:38.18 μg/mL) extracts; (B) Levels of UCK2 mRNA expressed in cells treated with FKB at 12.5 (3.55 μg/mL), 25 (7.1 μg/mL), and 50 μM (14.2 μg/mL); (C) Levels of UCK2 mRNA expressed in cells treated with APN at a concentration of 12.5 (3.37 μg/mL), 25 (6.75 μg/mL), and 50 μM (13.5 μg/mL). The housekeeping gene, GAPDH was used as loading control. C, Untreated control; D, DMSO used as negative control at a final concentration of 0.1%. (TIF) [file pone.0170233.s001.tif]

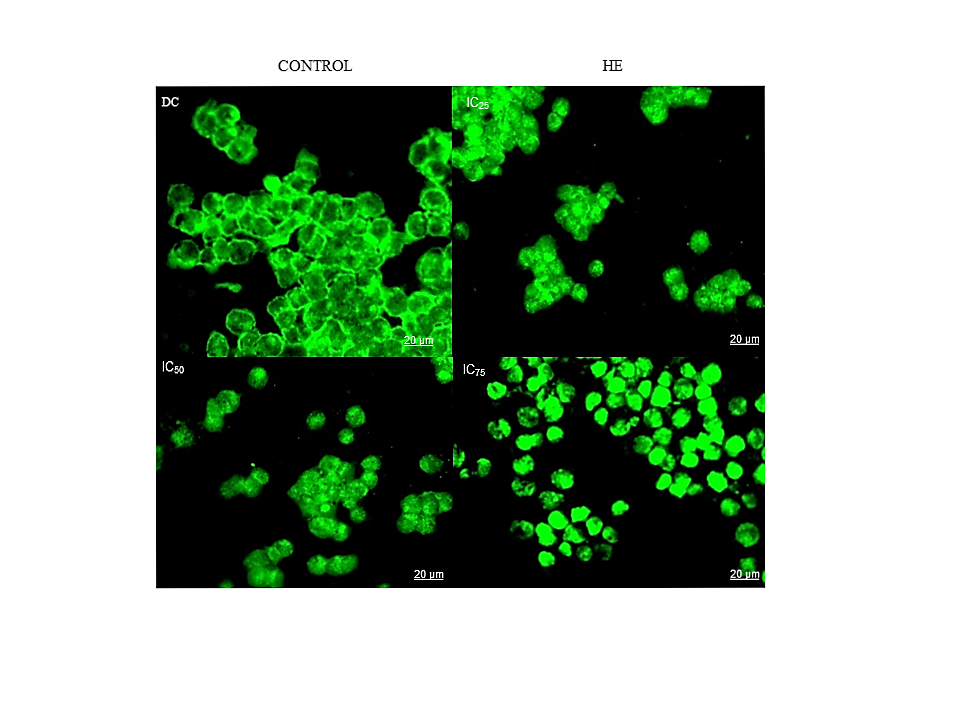

Supplement: S2 Fig — Cells were stained with AO and imaged using fluorescence microscope in exposure settings at 20× magnification. DC: DMSO treated control at a final concentration of 0.1%. (TIF) [file pone.0170233.s002.tif]

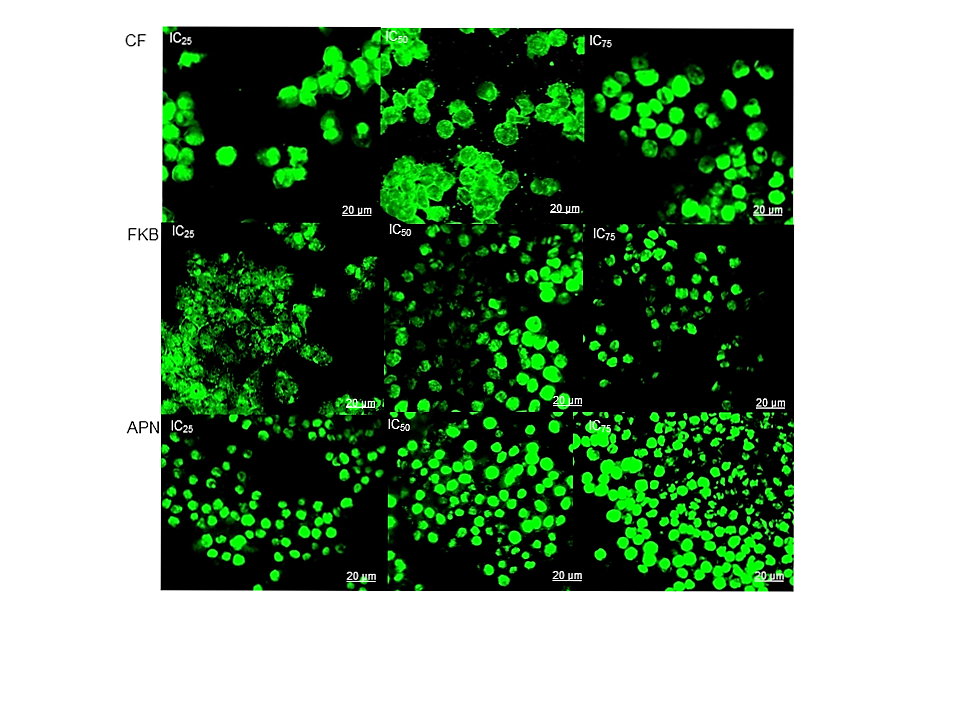

Supplement: S3 Fig — Cells were stained with AO and imaged using fluorescence microscope in exposure settings at 20× magnification. DC: DMSO treated control at a final concentration of 0.1%. (TIF) [file pone.0170233.s003.tif]
